# Intraoperative Radiotherapy in Brain Malignancies: Indications and Outcomes in Primary and Metastatic Brain Tumors

**DOI:** 10.3389/fonc.2021.768168

**Published:** 2021-11-11

**Authors:** Christopher P. Cifarelli, Geraldine M. Jacobson

**Affiliations:** ^1^ Department of Neurosurgery, West Virginia University, Morgantown, WV, United States; ^2^ Department of Radiation Oncology, West Virginia University, Morgantown, WV, United States

**Keywords:** IORT, brain metastases, glioblastoma, radiotherapy, local control, radiobiology

## Abstract

Despite the continued controversy over defining an optimal delivery mechanism, the critical role of adjuvant radiation in the management of surgically resected primary and metastatic brain tumors remains one of the universally accepted standards in neuro-oncology. Local disease control still ranks as a significant predictor of survival in both high-grade glioma and treated intracranial metastases with radiation treatment being essential in maximizing tumor control. As with the emergence and eventual acceptance of cranial stereotactic radiosurgery (SRS) following an era dominated by traditional radiotherapy, evidence to support the use of intraoperative radiotherapy (IORT) in brain tumors requiring surgical intervention continues to accumulate. While the clinical trial strategies in treating glioblastoma with IORT involve delivery of a boost of cavitary radiation prior to the planned standard external beam radiation, the use of IORT in metastatic disease offers the potential for dose escalation to the level needed for definitive adjuvant radiation, eliminating the need for additional episodes of care while providing local control equal or superior to that achieved with SRS in a single fraction. In this review, we explore the contemporary clinical data on IORT in the treatment of brain tumors along with a discussion of the unique dosimetric and radiobiological factors inherent in IORT that could account for favorable outcome data beyond those seen in other techniques.

## Introduction

The utility of adjuvant radiation in the management of high-grade primary central nervous system (CNS) tumors and intracranial metastatic disease is one of the few areas of modern neuro-oncology where consistent consensus opinions exist. Guideline recommendations from neurosurgical, radiation oncology, and medical oncology professional organizations repeatedly acknowledge the advantages in overall survival and progression-free survival times in populations treated with adjuvant radiation following surgical resection where surgery was deemed to be part of the standard of care (1, 2). Yet, beyond acceptance under the broad banner of “radiation treatment,” significant debates remain with respect to the modality of radiation delivery, dose, fractionation schedule, and time to initiation (3, 4).

With advancements in delivery techniques come the potential for improved outcomes, both in the areas of tumor control data and safety profiles. Over the past several decades, intraoperative radiotherapy (IORT) has emerged as one of those techniques with several studies demonstrating both feasibility and outcome equivalence, if not superiority when applied in the optimal setting. In the current review, we will examine the available clinical data on the use and applicability of IORT in CNS tumors, including metastatic disease and primary tumors. Moreover, we will discuss IORT within the broader context of intracranial brachytherapy, including the use of X-ray, electron, and radioisotope sources, all of which possess advantages and relative disadvantages in dose delivery.

## IORT Nomenclature

**Figure 1:**
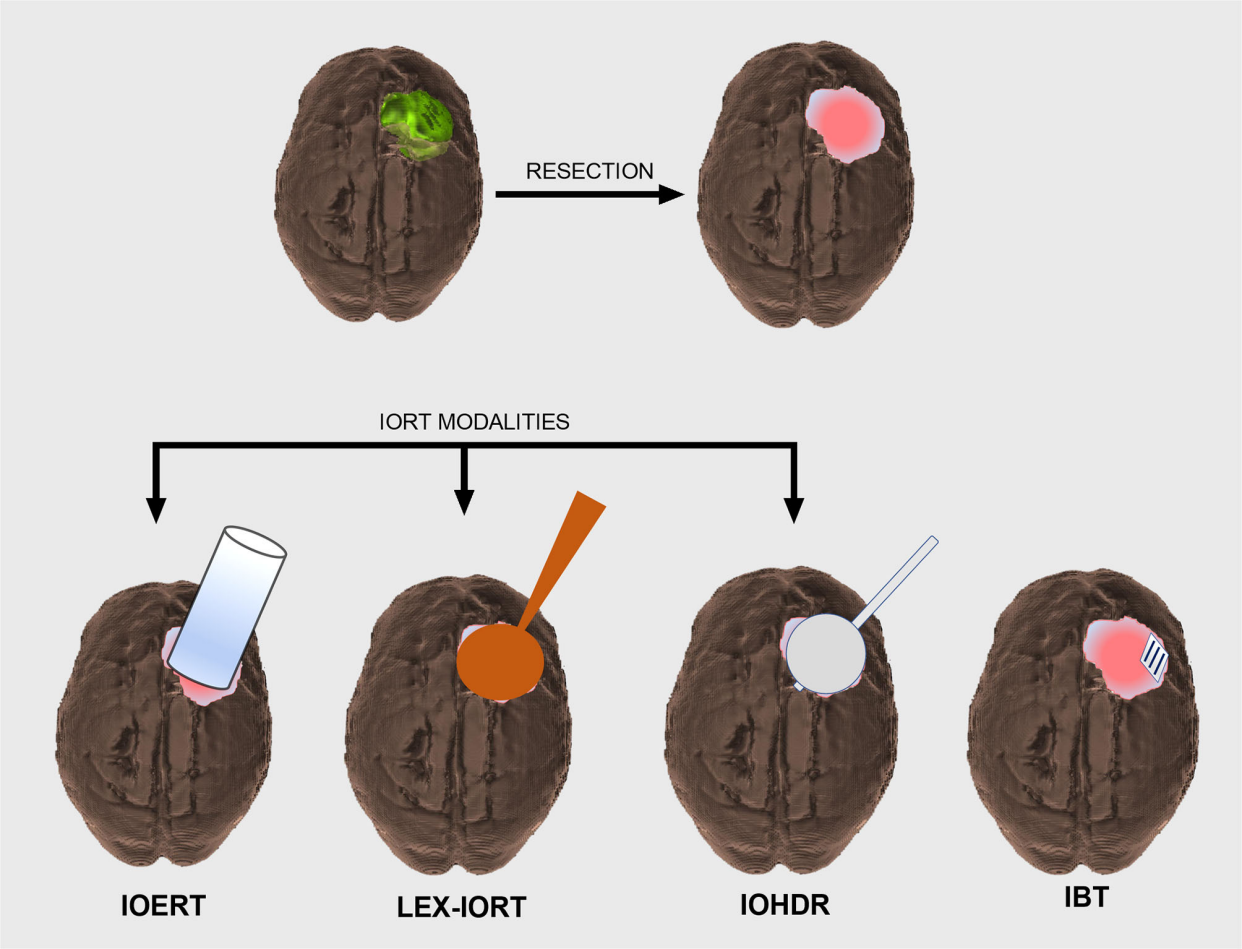
Schematic overview of CNS intraoperative radiotherapy (IORT) modalities. Intracranial tumor (*green*) prior to surgical resection resulting in a postoperative cavity that will require adjuvant radiation treatment (*top row*). IORT options include intraoperative electron radiotherapy (IOERT), low-energy X-ray intraoperative radiotherapy (LEX-IORT), and intraoperative high-dose rate brachytherapy (IOHDR) (*bottom row*). Interstitial brachytherapy (IBT) is distinct from IORT in that radiation dosing extends beyond the anesthetized surgical event.

In its simplest sense, IORT is defined as a single dose of radiation treatment delivered within the same anesthesia episode of care in which the tumor is resected or biopsied. Beyond this broad definition, several distinct treatment modalities exist under the umbrella of IORT with the primary differentiation between treatments being based on radiation source. A schematic representation of IORT applications is provided in **Figure 1**.

*Intraoperative electron radiotherapy* (*IOERT*) has been widely available and extensively studied in the context of extracranial disease sites (5–7). With its origins in clinical use dating back to the 1960s, IOERT has been demonstrated to be feasible and effective in local control of disease in breast, pancreas, soft tissue sarcomas, head and neck, uterine, and colorectal cancers (8, 9). Structural limitations of the applicator tube have restricted use to cavities with clear line-of-sight parameters, although the recently described potential to link IOERT delivery devices to electromagnetic surgical navigation systems could afford access to previously unattainable targets (10, 11). Despite decades of use in non-cranial sites, only a single institutional experience has been published describing intracranial use in high-grade glioma (12).

*Low-energy X-ray intraoperative radiotherapy* (*LEX-IORT*) uses a 30- to 50-kV isotropic X-ray source with either fixed diameter rigid spherical applicators (Intrabeam^®^, Carl Zeiss Meditec AG, Jena, Germany) or miniaturized X-ray source balloon applicators (Xoft^®^, San Jose, CA, USA). Allowing for a more conformal apposition to the resection cavity walls than the IOERT devices, the LEX-IORT devices exhibit a steep dose gradient with most of the dose delivered within 5–10 mm of the applicator surface. While the majority of clinical trial and outcome data using LEX-IORT have come from the management of breast cancer *via* the TARGIT studies, recent clinical trials have involved glioblastoma and more widespread use in surgically resected intracranial metastatic disease (13, 14).

*Intraoperative high-dose rate brachytherapy* (*IOHDR*), which relies on a sealed radionuclide source being placed within the tumor resection cavity, originated based on the need to treat residual tumor in areas not readily accessible with electrons *via* an IOERT applicator tube (15, 16). Distinct from other brachytherapy methodologies, the entirety of IOHDR treatment is delivered while the patient is anesthetized (17, 18). Utilized with regularity in rectal cancer, soft tissue sarcomas, and head and neck carcinomas, no published outcome studies exist detailing the use of IOHDR intracranially. While extensive data do exist with the use of intracranial expandable balloon-based brachytherapy using I-125 and Cs-131, these therapies do not qualify as “intraoperative,” nor did data from such studies suggest effectiveness in establishing local control over standard therapy (19, 20).

*Interstitial brachytherapy* (*IBT*), which is often included in the discussion of IORT, is, in fact, surgically aided radiotherapy rather than intraoperative radiation. Unlike IOERT or LEX-IORT, which completes the course of radiation exposure during the single episode of general anesthetic used for tumor resection, IBT delivers the majority of dose to the resection cavity in the days to weeks following implantation *via* a low-dose rate source material, such as Cs-131. The recent development of a permanent titanium encapsulated Cs-131 implant embedded in a resorbable collagen-based matrix (GammaTile^®^, GT Medical Technologies, Tempe, AZ, USA) has renewed interest in its use for recurrent high-grade tumors of the CNS (21). Clinical trials are currently enrolling patients using these Cs-131 implants under the heading of “surgically targets radiotherapy (STaRT)” (22).

## Clinical Outcome Data

### Metastatic Disease

Based on disease incidence and the nearly universal acceptance of the need for adjuvant radiation in brain metastases (BMs) that require surgical removal, the impact of IORT on metastatic disease management has significant potential. Early feasibility and outcome studies of 50 kV LEX-IORT employed the strategy of combining stereotactic biopsy with radiotherapy from a needle-tip applicator (23). The development of spherical applicators and the improved outcomes of patients undergoing gross total resection (GTR) over biopsy only have afforded the opportunity to examine the efficacy of LEX-IORT beyond safety and feasibility (24). In the first such study, Weil et al. provided retrospective analysis of 23 patients diagnosed with solitary brain metastases where 50 kV LEX-IORT was provided at a dose of 14 Gy at 2 mm depth from the applicator surface. With local control (LC) and distant brain failure (DBF) rate approximately 50%, LEX-IORT was considered equivalent to alternative strategies of stereotactic radiosurgery (SRS) and whole-brain radiotherapy (WBRT) (25).

Extending from this work, Vargo et al. reported a dose escalation feasibility study where the surface dose was increased to 30 Gy, in line with the available safety data from the Intraoperative Radiotherapy in Newly Diagnosed Glioblastoma (INTRAGO) I trial, where patients received a median dose of 3 0Gy at the applicator surfaces in newly diagnosed glioblastoma followed by an additional 60 Gy in standard-of-care external beam radiation therapy (EBRT). Developing comparisons between organs at risk (OAR) doses in IORT-treated patients and their theoretical SRS plans to achieve the same cavity margin dose, the authors determined that LEX-IORT was capable of boosting marginal dose beyond traditional SRS techniques (26).

A multi-institutional cooperative study on LEX-IORT in BMs has followed with participations from three international centers also participating the INTRAGO II study for newly diagnosed glioblastoma. In this cohort, 54 patients were treated with a median dose of 30 Gy at the applicator surface using the Intrabeam^®^ device (Carl Zeiss Meditec AG, Jena, Germany) with reported 1-year LC rate of 88% and overall survival (OS) of 73% (14). In comparison, the 1-year LC rate in SRS-treated metastatic surgical resection cavities in a contemporaneous randomized phase III study was only 72% (27). Subset analysis of these patients revealed an even higher 1-year LC rate of 94% for GTR *versus* 62% for subtotal resection (STR), indicating that the steep dose gradient of LEX-IORT was better suited for the management of microscopic residual disease rather than bulky residual disease. These data are in congruence with the original studies that utilized the needle biopsy approach rather than GTR with 1-year LC rates of 30%–50% (23, 28).

An expanded analysis of one of the three institutional cohorts recently provided follow-up with additional patients receiving LEX-IORT for resected BMs with a median marginal dose of 20 Gy. In that study, the 1-year LC rate was 84%, less than the 88% LC rate where the median margin dose was 30 Gy, indicating a small but discernable dose dependency on the tumor control rate. Of note, the 20-Gy margin dose study also reported a radiation necrosis (RN) rate of 2.5% compared with 7% in the larger study with a median dose of 30 Gy at the applicator surface (29). In comparison, historical rates of RN in single fraction radiosurgery treatments based on the 12-Gy volume (*V*_12_) have been shown to be 10% for a 5-cm^3^ *V*_12_ (30).

Data from these IORT studies on BMs are summarized in **Table 1**.

**Table 1:** Clinical outcome studies of IORT in brain metastases and glioma.

Metastatic Disease						
	IORT Modality	Number of patients	Median Dose (Gy)	Local Control Rate	Additional Adjuvant Radiation	Overall Survival(median months)
Weil et al. (25)	LEX-IORT	23	14 (2mm)	50% (1-yr)	SRS (n=7) WBRT (n=6) SRS+WBRT (n=2)	30
Cifarelli et al. (14)	LEX-IORT	54	30 (surface)	88% (1-yr)	SRS (n=1) WBRT (n=3)	na
Kahl et al. (29)	LEX-IORT	44	20 (surface)	84% (1-yr)	WBRT (n=10)	26.4
**High Grade Glioma**						
Schueller et al. (31)	IOERT	71	20 (90-100% isodose new diagnosis) 25 (90-100% isodose; recurrent)	4% (2-yr)	60 Gy	12.2
Usychkin et al. (12)	IOERT	32	12.5 (new diagnosis) 10 (recurrent)	na	46-60Gy	14 (new diagnosis) 10.4 (recurrent)
Sarria et al. (32)	LEX-IORT	51	30 (surface)	46% (1-yr)	60Gy	18

LEX-IORT, low-energy X-ray IORT; IOERT, intraoperative electron radiotherapy; SRS, stereotactic radiosurgery; WBRT, whole-brain radiotherapy; EBRT, external beam radiotherapy; na, not available.

### High-Grade Glioma

Radiotherapy has been a mainstay of treatment in high-grade glioma (HGG) and glioblastoma (GBM) for decades. Even in the absence of viable chemotherapeutic options based on comorbidities such as age, radiotherapy has remained a critical component of treatment (33, 34). In regard to the potential applications of IORT in the management of HGG and GBM, the consensus remains that its utility is in providing a boost to standard-of-care EBRT, usually in the recurrent/progressive disease setting (35). In the largest study of IOERT in HGG, Schueller et al. reported 71 total patients, 52 of which received a 20-Gy boost followed by 60-Gy EBRT for newly diagnosed tumors without significant improvement in OS or PFS intervals (31). Usychkin et al. provided an additional description of use of IOERT, both in newly diagnosed and recurrent glioma patients, including anaplastic astrocytoma, GBM, and anaplastic oligodendroglioma based on contemporaneous WHO classifications (12). Of the 32 patients treated, nearly half represented recurrent disease (47%) with a subtle dose reduction of a median 10 Gy for recurrent tumors from 12.5 Gy for newly diagnosed disease. Within the newly diagnosed patients, only six were GBMs with 15 of 17 patients having received postoperative EBRT ranging from 40 to 60 Gy in addition dose beyond the IOERT (12). Although the median OS for the entire study was 13 months, variability in histological diagnoses and natural disease progression diminishes the overall applicability of the data to HGG patients.

More recently, detailed phase I/II and phase III clinical trials have been designed and implemented for the analysis of IORT impact on newly diagnosed GBM exclusively using LEX-IORT. The INTRAGO clinical trials have established the safe dose range (20–30 Gy) for IORT in newly diagnosed GBM followed by 60-Gy EBRT according to standard-of-care treatment regimens (13, 36). While the phase III study continues to enroll newly diagnosed patients receiving LEX-IORT plus EBRT *versus* standard of care EBRT, a pooled analysis of INTRAGO LEX-IORT-treated patients and similarly treated patients from centers in Peru and China has been published. Representing a total of 51 patients, the median OS at 12, 24, and 36 months was 80%, 39%, and 26%, respectively. The overall PFS time was 11.4 months, with 12-, 24-, and 36-month rates of 46%, 29%, and 6%, respectively (32). Based on these preliminary findings, the final results of the phase III study (INTRAGO II) have been eagerly anticipated.

Data from these IORT studies on HGG are summarized in **Table 1**.

## Dosimetric Data

Given the predominant use of IORT as a means of providing a boost to more traditional external beam radiation treatments, the correct application of dosimetry measures is critical to both safety, in avoidance of adverse radiation effects or RN, and the efficacy of treatment. Dose depth gradients (DDG), homogeneity indices (HI), and relative biological effectiveness (RBE) have been shown to differ slightly among IORT modalities and even more so in comparison with other forms of radiotherapy and radiosurgery. As such, the characteristics of each should ideally be factored into the choice of modality for a given patient.

The steep dose gradient associated with IORT treatments can be advantageous or detrimental depending on primary treatment objectives. For local control in GTR metastatic tumor cavities, such gradients can provide maximal dose to non-visualized microscopic disease while minimizing risk to adjacent brain parenchyma. In the STR metastatic tumor, rapid dose decrements can allow for under treatment of residual disease, especially when IORT serves as the only adjuvant radiotherapy after surgery. The LC rate differences in GTR *versus* STR treated with LEX-IORT were clearly demonstrated by Cifarelli et al., with 1-year LC rate of 94% and 62%, respectively (14). In the situation of residual macroscopic tumor, the slightly greater depth-dose distribution provided by IOERT compared with LEX-IORT could prove to be beneficial. Once again, the balance of LC *versus* adverse effects attributed to radiation injury to normal tissue requires consideration.

The homogeneity of dose delivered also has the capacity to impact outcomes, necessitating some evaluation in the process of selecting an ideal IORT modality. While traditional measures of dose homogeneity are expressed as a function of maximal and minimal doses for a particular tumor volume, the cavitary targets treated in IORT add a layer of complexity to such calculations. In comparing the homogeneity of dose between IOERT and IOHDR, Calvo et al. reported that those delivered *via* IOERT were more evenly distributed across a given depth than those achieved by IOHDR (37). Beyond IORT modalities, homogeneity comparison between LEX-IORT and SRS has been performed, with evidence supporting superior homogeneity in LEX-IORT for single fraction treatment over that of SRS delivered by the Gamma Knife^®^ (Elekta AB, Stockholm, Sweden) (38).

Perhaps the best predictive measure of clinical outcomes following radiation treatment, regardless of modality, is the RBE. Several studies have examined the RBE of IORT modalities, noting specifically that the linear energy transfer (LET) of LEX-IORT produces more lethal macromolecular damage than IOERT (39–41). When tested directly in cultured tumor cell populations, the RBE across IORT, IBT, and EBRT energy sources varied based on cell line, indicating a more complicated radiation-induced cell death model than can be explained by stoichiometric differences in DNA damage (42). One such complicating factor includes the potential for radiation-induced bystander effect (RIBE) to increase the cell death following IORT modalities beyond that which would be predicted. Analysis of tumor cavity wound fluid after IORT in breast cancer patients indicates a persistent capacity to impact tumor cell proliferation, epithelial–mesenchymal transformation (EMT), and transcriptional regulation (43–45). Although representing a drastically different tumor microenvironment than that of the chest wall, the potential of cytokine release and immune modulation within the CNS has been hypothesized to play a role in potential IORT effects beyond radiation-induced DNA damage (46).

## Planning and Logistic Parameters

By its very nature, IORT is a team-based approach to patient care consolidated into a single episode. Coordination of the efforts of the surgical team, radiation oncologist, and physics support is a critical component of success and often a limiting factor when determining the feasibility of IORT as a viable treatment. Perhaps in neurosurgical oncology more than in other surgical oncology disciplines, procedures are often scheduled on an urgent rather than elective basis. Peritumoral edema, mass effect, and neurological symptoms all factor into the need for surgical resection in an expedited manner, leaving a short window for schedule coordination among the team members. Whereas the added benefit of a multidisciplinary approach to care is regularly touted as universal, with respect to IORT planning, this can be viewed as a logistic disadvantage (47, 48). The lack of availability based on concurrent clinical obligations by a single member of the team can be a significant factor that needs to be considered in the development of an IORT treatment strategy.

Beyond securing the team needed to successfully deliver an IORT treatment, feasibility needs to be established based on dose limitations for OARs and the ability to position an applicator device in the appropriate target area. Unlike IORT for breast or pelvic organs, the OARs within the CNS are capable of falling within an area of high dose, even with a steep dose gradient. Increased applicator size has been associated with significant increases in doses to the optic apparatus and brainstem in LEX-IORT where the diameter of the spherical applicator in LEX-IORT from the Intrabeam^®^ device exceeds 4 cm (26). Conversely, too little dose can be provided to the intended target if a close approximation of the applicator wall is lost (49). Residual bleeding and cerebrospinal fluid egress in the plane between the cavity wall and applicator are developments that the surgical team needs to be prepared to correct before deeming IORT to be technically feasible. To this end, not all intracranial tumors requiring surgical resection will be eligible for IORT. Hence, lesions that are amenable to GTR and at adequate distance from OAR structures are ideal candidates, although intraoperative judgment of the multidisciplinary team ultimately dictates IORT use.

One of the more obvious advantages of IORT over postsurgical radiation treatment can include elimination of the interval between surgery and radiotherapy or the time to initiation (TTI). Several groups have specifically identified increases in the TTI with decreased efficacy in attaining LC in brain metastases with SRS (50, 51). Potential causative factors for such treatment failures may include repopulation of tumor cells within the resection cavity walls and/or the difficulty of accurate target delineation in a resection cavity that has undergone postoperative remodeling (52, 53). In the complete elimination of the TTI and target cavity conformality *via* direct apposition to the tumor resection margin, IORT modalities may have the capacity to provide better outcomes than delayed radiotherapy approaches.

## Conclusions

Clinical applications for IORT continue to expand into the realm of neuro-oncology. While several studies have established the feasibility of using IORT as an adjunctive radiation treatment for primary CNS malignancies and metastatic disease, data from well-controlled clinical trials currently underway are likely to establish superiority of one modality relative to other forms of IORT or traditional SRS and EBRT techniques over time. While the unique dosimetry and timing of IORT has potential radiobiological advantages in achieving local control of resected CNS tumors, continued investigation in conjunction with novel systemic treatments will be needed in the future.

## Author Contributions

CC and GJ contributed to the composition and editing of the manuscript, including the table and figure. All authors contributed to the article and approved the submitted version.

## Conflict of Interest

CC has received speaking honoraria from Carl Zeiss Meditec AG for educational seminars.

The remaining author declares that the research was conducted in the absence of any commercial or financial relationships that could be construed as a potential conflict of interest.

## Publisher’s Note

All claims expressed in this article are solely those of the authors and do not necessarily represent those of their affiliated organizations, or those of the publisher, the editors and the reviewers. Any product that may be evaluated in this article, or claim that may be made by its manufacturer, is not guaranteed or endorsed by the publisher.
